# Association between Smokeless Tobacco Use and Risk of Periodontitis in Asian Countries: A Systematic Review and Meta-Analysis

**DOI:** 10.31557/APJCP.2021.22.10.3061

**Published:** 2021-10

**Authors:** Abhishek Mehta, Venkitachalam Ramanarayanan, Vineetha Karuveettil, Chandrashekar Janakiram

**Affiliations:** 1 *Department of Public Health Dentistry, Faculty of Dentistry, Jamia Millia Islamia, New Delhi, India. *; 2 *Department of Public Health Dentistry, Amrita School of Dentistry, Amrita Vishwa Vidyapeetham, India. *

**Keywords:** Smokeless tobacco, periodontitis, systematic review

## Abstract

**Background:**

Individual studies conducted in Asian countries have reported higher risk of periodontitis among smokeless tobacco (SLT) users in comparison to non-users. Therefore, a systematic review was conducted to summarize the available evidence on this topic.

**Methods:**

Prominent electronic databases were searched using pre-decided MeSH terms and keywords. Screening of titles and abstracts, full text reading, quality assessment and data extraction was done by two investigators independently. The Newcastle-Ottawa scale was used for risk of bias assessment of eligible studies. Meta-analysis was performed for four periodontal outcomes (periodontal pocket depth, loss of attachment, clinical attachment level and gingival recession). A sensitivity analysis was also performed.

**Results:**

Of the 546 citations, 367 were screened for eligibility. Finally, 89 studies were shortlisted for full text reading, of which, 36 were found eligible for qualitative analysis. Most of the studies were conducted in India (n=22), were of cross-sectional design (n=33), utilized purposive sampling and 24 studies were included for meta-analysis (n=28) and done on hospital-based population (n=26). Only 13 (37.1%) studies achieved a score of more than 50% (5/10 stars) on quality assessment scale. SLT users had higher odds of greater periodontal pocket depth greater than 4 mm (OR=3.64), gingival recession (OR=1.71) and loss of attachment 4-5 mm (OR=2.83) and mean difference of 1.7 mm for Clinical Attachment Level compared to non-users.

**Conclusion:**

The studies included in this review suggests that SLT users have poorer periodontal health in comparison to non-users. But most of this evidence comes from cross-sectional studies. Longitudinal studies with rigorous methodology are required to support this elucidation.

**Registration:**

This systematic review protocol has been registered in PROSPERO (CRD42019122964).

## Introduction

Periodontal disease is an inflammatory response of the periodontium to toxins released by bacteria present in plaque bio-film. It is broadly characterized into two conditions, gingivitis (reversible inflammation of gums) and periodontitis (destruction of periodontal ligament and alveolar bone). Severe periodontitis is the 6^th^ most common disease and is the leading cause of multiple tooth loss around the world (Jin et al., 2016). Evidence suggests that smoking tobacco (Leite et al., 2018), poorly controlled diabetes mellitus (Mauri-Obradors et al., 2017) and poor oral hygiene (Lertpimonchai et al., 2017) are major risk factors for periodontitis. Male gender, advanced age, and poor socioeconomic status are other factors that are found to be associated with periodontitis (Eke et al., 2012). These three factors are also found to be associated with tobacco consumption (Leite et al., 2018; Thakur and Paika, 2018).

The role of tobacco use, especially in smoking form, is studied extensively in the etiology of periodontitis. Recently, a meta-analysis of longitudinal studies had reported smoking tobacco increased the risk of periodontitis by 85% (risk ratio 1.85, 95% CI 1.5, 2.2)(Leite et al., 2018). The other form of tobacco i.e. smokeless tobacco (SLT) has received less attention of researchers, hence the association between SLT use and periodontal disease is still unclear(Kamath et al., 2014). There are many reasons for this research lacuna such as availability of many types of SLT products in different parts of the world, addition of other ingredients to SLT, and variation in quantity and frequency of consumption (Critchley and Unal, 2003; Siddiqi et al., 2015). Unlike cigarette, SLT products are available and consumed in different forms, this non-standardization causes difficulty in studying association between their use and health conditions. 

We found two systematic reviews which were conducted to analyze the evidence on association between SLT use and periodontitis. Both these reviews included studies conducted in US and European countries (Critchley and Unal, 2003; Kallischnigg et al., 2008). These reviews had concluded that there is limited evidence available on the relationship between SLT use and periodontitis. They also highlighted the differences in SLT products used in western countries and Asian countries especially south and south-east Asia. The South Asian region alone is home to 90% (more than 250 million) of all SLT users globally (Sinha et al., 2012). The SLT used in South Asian countries is mostly chewing tobacco which is mixed with other ingredients such as betel quid, areca nut, paan masala, and slaked lime (Kamath et al., 2014). The usage of SLT is increasing worldwide especially in South Asian countries such as India and Bangladesh (11). Many factors are postulated for this trend such as cultural acceptability, low comparative cost and ease of purchase, and myth of medicinal use of SLT (Kathiriya et al., 2016). The increased consumption of SLT usage will amplify its deleterious health effects on the human population. The consumption of these SLT products has been found to increase the risk of oral cancers and oral potentially malignant disorders (Asthana et al., 2019; Khan et al., 2017). Although, studies are reporting a higher risk and severity of periodontitis among SLT users, there is a need to systematically analyze the quality of evidence available (Anand et al., 2013; Parmar et al., 2008). Therefore, we conducted a systematic review with an aim to summarize the evidence available on the use of SLT and its risk of causing periodontitis as compared to non-users among Asian population.

## Materials and Methods

This systematic review is conducted based on Preferred Reporting Items for Systematic Reviews (PRISMA) guidelines and registered in PROSPERO register of Systematic Reviews in March 2019 (Registration No.: CRD42019122964). The focused review question is “whether there is an association between smokeless tobacco consumption and increased risk of periodontitis as compared to non-users among the population of Asian countries?” The Population, Exposure, Comparator, and Outcome (PECO) applicable to this review are:

Population: Individuals living in countries of the Asian continent.

Exposure: Individuals consumed SLT ever in their life

Comparator: Individuals who had never consumed any form of tobacco.

Outcome: Measurement of presence and severity of periodontitis.

*Eligibility Criteria*

*Type of studies*

Original published observational studies (cross-sectional, case-control, cohort) that investigated the association between smokeless tobacco consumption and periodontitis in the human population residing in Asian countries were considered. Studies evaluating the effect of post periodontal therapy healing among SLT users were not included. Those studies where study participants were consuming smoking or both forms of tobacco were excluded. Studies in which participants used inhaled forms of tobacco or only arecanut, betel nut or any other chewing substance without addition of tobacco were not included in this review. Also not included were studies conducted outside Asia or on expatriate populations of Asia. The reasons were differences in composition of SLT products available in their current resident countries. The lifestyle of these expat populations might also differ from the population of their native countries (Khan et al., 2017). Additionally, animal studies, case reports, literature reviews and in vitro studies were excluded.

In a pre-review scoping exercise (unpublished) it was observed that there is a deficiency of prospective cohort studies or longitudinal analytical studies on this topic. Previous systematic reviews conducted on assessing health effects of SLT have reported similar issues (Critchley and Unal, 2003; Kallischnigg et al., 2008). Therefore, we focused on observational studies (of any type) to establish epidemiological association between SLT use and periodontitis.

*Exposure and outcome measurements*

Studies which assessed SLT exposure of an individual through hospital records, interview or self –reported questionnaire and where SLT use was the main exposures were included in the review. The criteria to assess periodontitis as stated in the studies was acceptable because the definition for assessing presence of periodontitis has undergone dynamic changes multiple times in the past (Savage et al., 2009). A person was considered SLT user if they give a history of consuming any form of SLT “ever” in their life. The comparison or controls were the individuals who told they had “never” consumed any form of tobacco.

*Search strategy*

A systematic electronic search was conducted in PubMed/Medline, Scopus, Open J gate, and Web of Science databases. We also searched the first 20 pages of Google Scholar. Reference lists of papers included for full text reading were hand searched. No language or date filters were applied. Studies published upto and including January 2020 were searched. Initial search was performed on PubMed using combination of these MeSH terms or keywords: Smokeless tobacco [MeSH] OR chewing tobacco for exposure and periodontal diseases [MeSH} OR gingivitis OR periodontitis [all] OR attachment loss [all] OR probing depth [all] OR tooth loss [all] OR gingival recession [all] for outcome.

*Studies selection*

The software Covidence (Covidence systematic review software, Veritas Health Innovation, Melbourne, Australia. Available at www.covidence.org) and Mendeley were used to manage the references at all stages. Removal of duplicate references was followed by evaluation of the title and abstracts of the remaining articles by the two investigators (AM and RV) independently. After applying eligibility criteria, eligible articles were shortlisted for full text reading by both the investigators. In case of disagreement, a consensus was reached through discussion with the third author (VK). 

*Data extraction*

We grouped the information extracted from eligible studies into the following categories:

*Publication*

Author and year of publication

*Study*

Design (cross-sectional, case-control) sample size (total, in case and comparison group), sampling technique used (probability or purposive), geographic location (country name), and type of population (general, hospital based, any other e.g. industrial workers).

*Exposure and outcome characteristics*

Type of SLT used and definition and criteria used to record periodontitis and SLT usage. Both the investigators (AM and RV) had extracted the data independently. Expert opinion of the third investigator (VK) was taken in case of disagreement and consensus was reached.

*Critical appraisal of studies/quality assessment*

The quality appraisal of case-control studies was done using a specific version of Newcastle-Ottawa scale (Wells et al., 2011) and for cross-sectional studies an adapted version of the same scale was used (Modesti et al., 2016). A meeting of all the reviewers was conducted to agree upon how each parameter of the quality assessment scale should be evaluated. An overall score for each study was calculated and they were then assigned into categories of low, moderate and high methodology quality.

*Statistical analysis*

The quantitative synthesis was done for four periodontal outcomes commonly reported across the studies; viz. periodontal pocket depth (PPD), clinical attachment level (CAL), los of attachment (LOA) and gingival recession (GR). The summary statistical measure used was mean difference for continuous outcomes (PD, CAL, GR) and Odds ratio for binary outcomes (proportion of people with PD > 4mm, PD > 6mm, LOA 4-5 mm, LOA > 6 mm and GR). Heterogeneity was assessed using I2 statistics. An I2 value of heterogeneity of the data using Cochran’s Q statistic, a chi-square test, a threshold p-value of less than 0.10. The consistency of the results was assessed visually using forest plots and by the I2 statistic (Higgins and Thompson, 2002). Random-effects models (add ref) were used to calculate a pooled estimate of effect and the summary measure was reported with 95% confidence intervals (CIs). A sensitivity analysis was performed for studies that reported wide variations in outcomes. 

## Results

**Figure 1:**
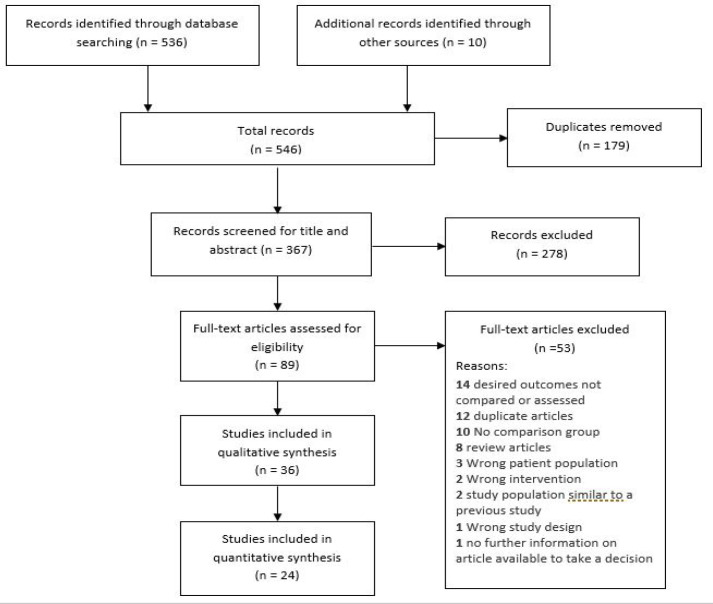
PRISMA Flowchart

Electronic search retrieved 546 records from the four databases (PubMed, Web of science, open J-gate and Scopus; n=536), reference lists (n=8) and Google Scholar (n=2). Duplicate records (n=179) were removed using Covidence and Mendeley software. Abstract and titles were screened for 367 citations based on the inclusion and exclusion criteria. From those, 89 articles were extracted which had full-text availability. We excluded 53 articles as they were found not eligible for inclusion in this review. Finally, 36 studies were included for qualitative synthesis and 24 studies were included for meta-anlaysis (Figure 1).

*Qualitative synthesis*

We listed the main characteristics of the included studies as study design, sample population (case, control), Smokeless tobacco (type, exposure), definition of exposure, definition of disease and quality assessment score (Table 1). Considering study design, three were case- control studies (Akhter et al., 2008; Kalburgi Nagaraj, 2013; Wellapuli and Ekanayake, 2017) and 33 studies adopted cross-sectional study design. The studies investigated different forms of tobacco ranging from Betel quid with tobacco to Gutka, Shamma, Mishri, Gutaku and Naswar. All the studies had included current SLT users for comparison of periodontal findings with non-chewers and only four studies included former SLT users. Smokeless tobacco exposure was assessed based on duration and frequency of SLT use. Majority of the studies (n=14) assessed smokeless tobacco use of participants at the time of the study, while others had time frames of at least a week, 3 months, 1 year, 2 years to a maximum of 5 years. Consuming tobacco once daily was considered as a criterion for exposure for the majority (n=11) of the studies while few required a frequency of 4 times to 10 times daily as a criterion. When defining the periodontitis, it is important to note that 28 studies didn’t utilize a proper definition of the disease in question. Two studies (Jacob et al., 2014; Wellapuli and Ekanayake, 2017) used CDC (Centre for Disease Control and Prevention) definition of periodontitis. While probing depth and Clinical loss of attachment were used to define the disease by three studies (Akhter et al., 2008; Mittal et al., 2017; Mohamed and Janakiram, 2013) . Marginal bone loss and CPI index was used by Kalburgi NB et al. 2014 (Kalburgi Nagaraj, 2013) and Baishya B et al. 2019 (Baishya et al., 2019) respectively to define the disease in question. Quality appraisal scores of the studies ranged from 2 to 6 (Akhter et al., 2008; Amarasena et al., 2002; Kumar et al., 2008; Mahapatra et al., 2018; Mohamed and Janakiram, 2013). A positive finding in our systematic review is defined as the presence of a significant association between smokeless tobacco and periodontal disease. Work done by Kulkarni et al., (2016) and Nagarajappa and Prasad (2010) reported no significant association between exposure and outcome. All the other studies had positive findings for all the parameters establishing significant association between SLT use and occurrence of periodontitis (Table 1). 

Periodontal, gingival and other parameters related to periodontal conditions were measured in the studies. LOA was measured in 22 studies to measure periodontitis. PPD was assessed by 21 studies and radiological assessment of Marginal Bone loss was performed by three studies (Al-Askar et al., 2017; Javed et al., 2013b; Javed et al., 2015). Periodontal indices used were Community Periodontal Index (10 studies), Community periodontal Index of Treatment Needs in two studies (Jacob et al., 2008; Sharma and Oberoi, 2018) and Periodontal Disease Index (Kulkarni et al., 2016). Gingival recession (GR) was assessed in seven studies (Anand et al., 2013; Jacob et al., 2014; Mittal et al., 2017; Muhammad Yasir Ilyas et al., 2015; Parmar et al., 2008; Singh et al., 2011; Verma et al., 2019). Along with GR, two studies assessed mobility and furcation involvement among SLT users (Singh et al., 2011; Verma et al., 2019). Gingival parameters adopted included bleeding on probing (12 studies) and Gingival Crevicular Fluid Measurement (Mittal et al., 2017). Amarasena et al., (2002) and Amarasena et al., (2002) used levels of bacterial plaque (PLI) and Gingival inflammation (GI) to measure gingival condition. Indices adopted to measure gingival condition were Plaque Index (13 studies), gingival Index (7 studies) and calculus index (2 studies). Oral hygiene status was measured using Oral Hygiene Index - Simplified in seven studies (Arun Kumar et al., 2012; Ashwini et al., 2014; Giovannoni et al., 2018; Kulkarni et al., 2016; Parmar et al., 2008; Ravishankar PL et al., 2017; Verma et al., 2019). Confounding factors were considered and adjusted only in few studies (Akhter et al., 2008; Al-Tayar et al., 2015; Javed et al., 2013a; Wellapuli and Ekanayake, 2017). The factors included were Sociodemographic characteristics (age, gender, occupation), use of smoking form of tobacco, body mass index, stress, dental visit pattern, oral hygiene habits and plaque Index. Since the long-term progression of periodontal disease is tooth loss, three studies (Akhter et al., 2008; Javed et al., 2015; Muhammad Yasir Ilyas et al., 2015) had measured edentulousness of the sample population. Mahapatra et al., (2018) used WHO Oral health assessment form to collect details on periodontal, gingival and edentulousness of SLT users. There were only 10 studies that reported measures of association between SLT users and non-users with periodontitis in the form of Odds Ratio (Akhter et al., 2008; Al-Tayar et al., 2015; Giovannoni et al., 2018; Javed et al., 2013a; Kathiriya et al., 2016; Mahapatra et al., 2018; Mohamed and Janakiram, 2013; Parmar et al., 2008; Singh et al., 2011; Wellapuli and Ekanayake, 2017) (Table 2).

*Quantitative synthesis*

Risk difference between the SLT and non-SLT groups for chronic periodontitis was assessed based on four clinical parameters –PPD, GR, LOA and CAL. The forest plots were prepared based on the results obtained from application of random-effect model. A random effects model was chosen to account for the clinical and methodological heterogeneity. 

**Figure 2:**
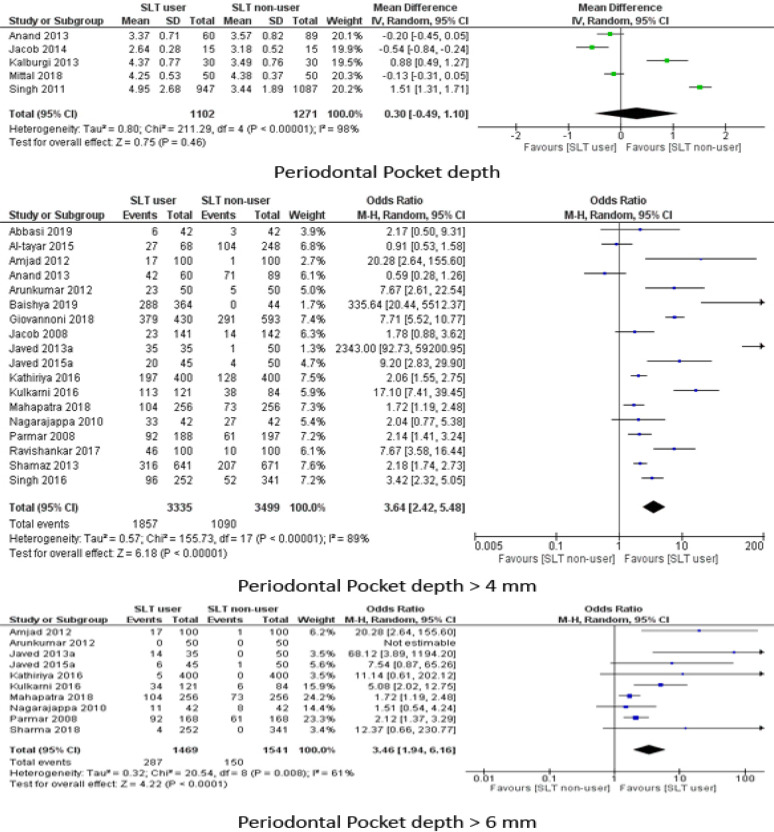
Meta Analysis of Pocket Depth (Mean and proportion)

**Figure 3:**
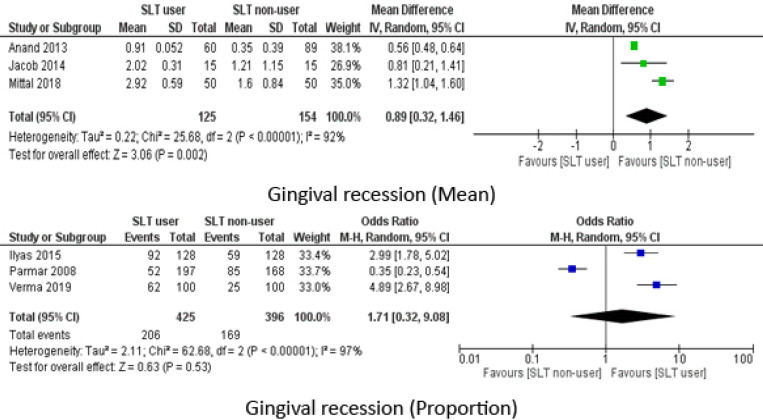
Meta Analysis of Gingival Recession (mean and proportion)

The ORs for PPD > 4mm (18 studies) and PPD > 6 mm (10 studies) was [OR: 3.64 (95% CI: 2.42, 5.48)] and [OR: 3.46 (95% CI: 1.94, 6.16)] respectively for SLT group in reference to non-users. I^2^ was higher for studies comparing PPD > 6mm (89%) compared to PPD > 4mm (61%), although, it was statistically significant in both cases. The mean PPD was slightly higher for SLT group [MD: 0.30 (95% CI:0.49, 1.10)] as shown in Figure 3. A sensitivity analysis was performed for the outcome PPD > 4mm as some four studies had very high odds ratio. After excluding four studies, the OR was more than twice higher in the SLT group compared to non-users [OR: 2.57 (95% CI: 1.79, 3.70)] (Figure 2).

Gingival recession was reported by six studies either as presence/absence of the condition or as mean values. The calculated OR was 1.71 (95% CI: 0.32, 9.08) with non-users being the reference category and mean recession levels were greater for SLT group (MD: 0.89 95% CI: 0.32, 1.46). The heterogeneity was significantly high as I2 was above 90% (Figure 3).

**Figure 4:**
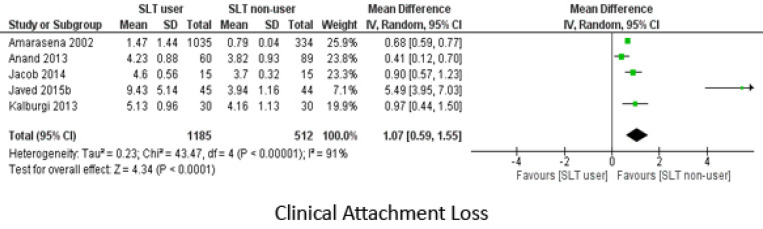
Meta Analysis of Clinical Attachment Loss (Mean)

**Figure 5:**
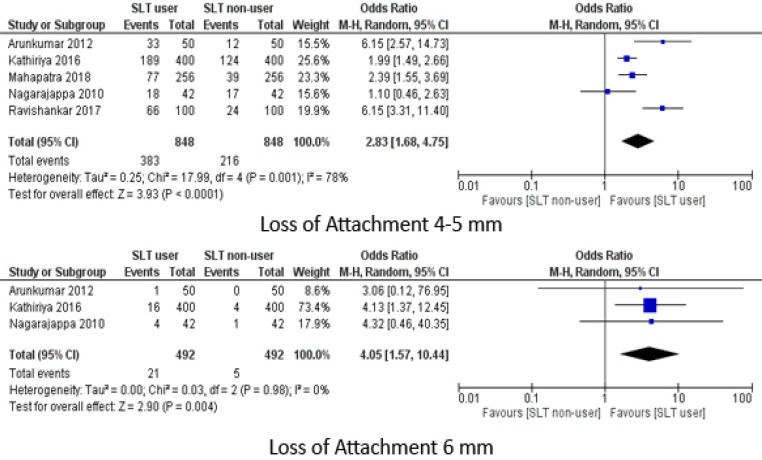
Meta Analysis of Loss of Attachment (Proportions)

Differences in the CAL between the two groups was calculated from data obtained from five studies and mean difference was 1.7 (95% CI: 0.57, 1.55) (Figure 4). For Loss of Attachment (LOA) outcome which was commonly reported in proportions, OR was 2.83 (95% CI: 1.68,4.75) and 4.05 (95% CI: 1.57, 10.44) for LOA of 4-5 mm and 6 mm respectively. Significant level of heterogeneity was observed between the studies when calculating mean difference of CAL and LOA of 4-5 mm but not for LOA of 6 mm (Figure 5). All these calculated values signifies that SLT users had poorer periodontal condition as compared to non-SLT group.

**Table 1:** Characteristics of Included Studies

Source	ST use		Type of population	Type of sampling	Definition of Exposure	Definition of Disease	QA Score
	Type	Exposure					
Case Control studies							
Akhter 2008 (Akhter et al., 2008) (Bangladesh)	BQ with Tobacco	Current, Former users	Hospital-based	Convenience	SLT use status at time of study	Yes, PD>5 or CAL >6	6
Kalburgi 2014 (Kalburgi et al., 2014) (India)	Not mentioned	Current	Hospital-based	Convenience	SLT use for at least 15 years	Yes, Chronic Periodontitis PD>=6 IN 30% sites and MBL> 50%	2
Wellapuli 2017 (Wellapuli and Ekanayake, 2017)	BQ with Tobacco	Current	Record-based	Convenience	SLT use status at time of study	CDC def of mod and severe periodontitis	4
Cross-sectional studies							
Abbasi 2019 (Abbasi et al., 2019) (Pakistan)	Naswar	Current	Hospital-based	Convenience	SLT use >=12 months at least once daily	No	3
Al-askar 2017 (Al-Askar et al., 2017) (Saudi Arabia)	Gutka, Shamma	Current users	Hospital-based	Convenience	SLT use >=12 months at least once daily	No	5
Al-tayar 2015 (Al-Tayar et al., 2015) (Yemen)	Shamma	Current, Former users	General	Probability	SLT use status at time of study	No	5
Amarasena 2002 (Amarasena et al., 2002) (Sri Lanka)	BQ with tobacco	Current	General	Probability	SLT use >=12 months at least once daily	No	6
Amjad 2012 (Faiza Amjad et al., 2012) (Pakistan)	Not mentioned	Current	Hospital-based	Convenience	SLT use status at time of study	No	2
Anand 2013 (Anand et al., 2013) (India)	Not mentioned	Current	Hospital-based	Convenience	SLT use =5 years at least 10 times daily	No	3
Arunkumar 2012 (Arun Kumar MS et al., 2012) (India)	Gutka	Current	Hospital-based	Convenience	SLT use =3 months at least 4 times daily	No	2
Baishya 2019 (Baishya et al., 2019) (India)	Not mentioned	Current	General	Probability	SLT use status at time of study	Yes, Periodontitis if CPI score>2	5
Biradar 2014 (Ashwini SB et al., 2014) India)	Not mentioned	Current	Hospital-based	Convenience	SLT use status at time of study	No	3
Giovannoni 2018 (Giovannoni et al., 2018) (India)	BQ with tobacco; Tobacco alone	Current	Hospital-based	Convenience	SLT use at least 2 years	No	5
Ilyas 2015 (Muhammad Yasir Ilyas et al., 2015) (Pakistan)	Not mentioned	Current	Hospital-based	Convenience	SLT use status at time of study	No	2
Jacob 2008 (Jacob et al., 2008) (India)	BQ with tobacco; Tobacco alone	Current	Hospital-based	Convenience	SLT use status at time of study	No	3
Jacob 2014 (Jacob et al., 2014) (India)	Gutka	Current	Hospital-based	Convenience	SLT use >=12 months at least once daily	Yes, CDC def of moderate periodontitis	4
Javed 2008 (Javed et al., 2008) (Pakistan)	Gutka	Current	Hospital-based	Convenience	SLT use >=12 months at least once daily (one sachet)	No	4
Javed 2013a (Javed et al., 2013a) (Pakistan)	Gutka	Current	Hospital-based	Convenience	SLT use >=12 months at least once daily	No	3
Javed 2013b (Javed et al., 2013b) (Pakistan)	BQ with tobacco	Current	Hospital-based	Convenience	SLT use >=12 months at least once daily	No	4
Javed 2015a (F. Javed et al., 2015) (Pakistan)	Gutka	Current	Hospital-based	Convenience	SLT use >=12 months at least once daily	No	5
Source	ST use		Type of population	Type of sampling	Definition of Exposure	Definition of Disease	QA Score
	Type	Exposure					
Cross-sectional studies							
Javed 2015b (Fawad Javed et al., 2015) (Pakistan)	Gutka	Current	Hospital-based	Convenience	SLT use >=12 months at least once daily	No	2
Kalburgi 2013 (Kalburgi Nagaraj, 2013) (India)	Gutka	Current	Hospital-based	Not mentioned	SLT use for at least 5 year	Yes	3
Kathiriya 2016 (Kathiriya et al., 2016) (India)	Multiple	Current	Industrial workers	Probability	SLT use >=12 months at least once daily	No	5
Kulkarni 2016 (Kulkarni et al., 2016) (India)	Gutka; Mishri	Current	Hospital-based	Convenience	SLT use daily for at least 2 years	Yes, mild, moderate and severe periodontitis	4
Mahapatra 2018 (Mahapatra et al., 2018) (India)	BQ with tobacco; Gutka	Current	Hospital-based	Convenience	SLT use >=12 months at least once daily	No	6
Mittal 2017 (Mittal et al., 2017) (India)	Not mentioned	Current	Hospital-based	Convenience	SLT use for at least 2 years	Yes, at least 4 teeth with PD>3 OR CAL>1-3	4
Nagarajappa 2010 (Nagarajappa and Prasad, 2010) (India)	Multiple	Current	Hospital-based	Convenience	SLT use for at least 1 week	No	4
Parmar 2008 (Parmar et al., 2008) (India)	BQ with tobacco	Current	Hospital-based	Convenience	SLT use status at time of study	No	2
Philip 2013 (Biju Philip et al., 2013) (India)	Not mentioned	Current	Tribal	Probability	SLT use status at time of study	No	5
Rajkarnikar 2015 (Rajkarnikar and Acharya, 2014) (Nepal)	Not mentioned	Current	Hospital-based	Convenience	SLT use status at time of study	No	2
Ravishankar 2017 (Ravishankar PL et al., 2017) (India)	Gudakhu	Current	Hospital-based	Convenience	SLT use =3 months at least 4 times daily	No	2
Shamaz 2013 (Mohamed and Janakiram, 2013) (India)	Multiple	Current	General	Probability	SLT use status at time of study	Yes, CAL>4mm at least one site	6
Sharma 2018 (Sharma and Oberoi, 2018) (India)	Not mentioned	Current	Construction workers	Convenience	SLT use status at time of study	No	3
Singh 2011 (Singh et al., 2011) (India)	Multiple	Current, Former users	Hospital-based	Convenience	SLT use status at time of study	No	2
Singh 2016 (Singh et al., 2016) (India)	Not mentioned	Current	Police Personnel	Probability	SLT use status at time of study	No	5
Verma 2019 (Verma et al., 2019) (India)	Gutka	Current	General	Convenience	SLT users> 6 months at least 2 packets daily	No	2

**Table 2:** Clinical Assessment of Periodontal Disease among SLT Users and Measure of Association

Source	Periodontal parameters assessed	Cases	Controls	Summary of main statistical findings	Variables adjusted in statistical analysis
Case-control studies					
Akhter 2008 (Akhter et al., 2008) (Bangladesh)	PD, CAL	117	59	"Adjusted OR CAL [3.97* (1.19 – 13.30)]"	Gender, smoking habit, body mass index, dental visit pattern, education level, stress, and plaque index.
Kalburgi 2014 (Kalburgi et al., 2014) (India)	PD, CAL	30	30	"Mean scores PD - Control: 5.5 ± 0.57 - Gutka: 5.2 ± 0.41 CAL - Control: 6.1 ± 0.86- Gutka: 6 ± 0.74 "	
Wellapuli 2017 (Wellapuli and Ekanayake, 2017) (Sri Lanka)	PD, CAL	111	583	"Unadjusted OR PD [3.17* (2.17 – 4.63)] Adjusted OR PD [2.05* (1.34 – 3.14)]"	Gender, age, ethnicity, education level, monthly income, occupation, oral hygiene habits, alcohol
Cross-sectional studies					
Abbasi 2019 (Abbasi et al., 2019) (Pakistan)	PD, CAL	42	42	"PD* > 4mm - Naswar: 14.4% - Controls: 7% CAL* [p < 0.001]"	
Al-askar 2017 (Al-Askar et al., 2017) (Saudi Arabia)	PD, CAL, MBL (Radiographical)	41	92	"Mean difference CAL* - (SC Vs Control): 3.5 (2.6-4.4) - (GC Vs Control): 3.3 (2.5-4.1) MBL* - (SC Vs Control): 2.9 (1.6-5.0) - (GC Vs Control): 2.4 (13.5-26.1) PD 4-6mm* - (SC Vs Control): 19.6 (10.2-23.3) - (GC Vs Control): 24.1 (20.3-44.5) PD >6mm* - (SC Vs Control): 15.6 (1.6-5.0) - (GC Vs Control): 18.2 (13.5-26.1)"	
Al-tayar 2015 (Al-Tayar et al., 2015) (Yemen)	CPI	68	248	"Current Shammah user Adjusted OR - CPI* [6.62 (3.59 - 12.21)]"	Socio demographic characteristics and oral hygiene habits
Amarasena 2002 (Amarasena et al., 2002) (Sri Lanka)	CAL	334	1035	"CAL* - Betel Chewer: 1.47±1.44 - Non-users: 0.79±0.04"	
Amjad 2012 (Faiza Amjad et al., 2012) (Pakistan)	PD	100	100	"PD* > 6mm [p < 0.001] - User: 17/100 - Non-user: 1/100"	
Anand 2013 (Anand et al., 2013) (India)	PD, CAL, GR	60	89	"PD - ST users: 3.37± 0.71 - Never users: 3.57±0.82 GR*- ST users: 0.91±0.52 - Never users: 0.35±0.39 CAL* - ST users: 4.23±0.88 - Never users: 3.82 ± 0.93"	
Arunkumar 2012 (Arun Kumar MS et al., 2012) (India)	CPI, LOA	50	50	"CPI* [p = 0.0001]LOA* [p = 0.0001]PD > 4 mm- User: 23/50- Non-user: 5/50"	
Baishya 2019 (Baishya et al., 2019) (India)	CAL, CPI	364	44	"CPI score >2 [p<0.001]* - User: 288/364- Non-user: 0/44"	
Biradar 2014 (Ashwini SB et al., 2014) (India)	CPI, LOA	27	224	"CPI [p > 0.05] LOA [p > 0.05]"	
Giovannoni 2018 (Giovannoni et al., 2018) (India)	CPI	430	593	"Unadjusted OR CPI* [7.71 (5.5–10.77)] CPI > 3mm - User: 379/430 -Non-user: 291/593"	
Cross-sectional studies					
Ilyas 2015 (Muhammad Yasir Ilyas et al., 2015) (Pakistan)	GR	128	128	GR*: [p < 0.01]	
Jacob 2008 (Jacob et al., 2008) (India)	CPITN	141	142	CPITN > 3* [p < 0.001]- Connsumer: 23/141 - Non-consumers: 14/142	
Jacob 2014 (Jacob et al., 2014) (India)	PD, CAL, GR	15	15	Mean scoresPD*- Non gutka user [3.18 ± 0.52]- Gutka user [2.64 ± 0.28]CAL*- Non gutka user [3.70 ± 0.32]- Gutka user [4.60 ± 0.56]GR*- Non gutka user [1.21 ± 1.15]- Gutka user [2.02 ± 0.31]	
Javed 2008 (Javed et al., 2008) (Pakistan)	PD	36	42	Mean scorePD (>6mm) [p > 0.05]	
Javed 2013a (Javed et al., 2013a) (Pakistan)	PD, Periodontal inflammation	44	44	Odds RatioPI [1.6 (0.54 to 4.7)]Mean differencePD (>6mm)* [3.2 (0.7 – 5.7)]	Age, sex, gutka chewing, prediabetes, and (gutka · prediabetes) interaction.
Javed 2013b (Javed et al., 2013b) (Pakistan)	PD, MBL	35	50	PD* [p < 0.05]- User: 35/35- Non-user: 1/50MBL* [p < 0.01]	
Javed 2015a (F. Javed et al., 2015) (Pakistan)	PD, CAL, MBL	45	50	Mean difference PD (> 4mm)- User: 20/45- Non-user: 4/50PD* (>6mm)- GC vs NC [12.3 (7.1 – 16.3)]CL*- GC vs NC [3.7 (2.8 – 4.4)] MBL*- GC vs NC [2.6 (1.8 – 2.9)]	
Javed 2015b (Fawad Javed et al., 2015) (Pakistan)	PD, CAL	45	45	PD (>3mm)* [p < 0.01]CAL* [p < 0.01]	
Kalburgi 2013 (Kalburgi Nagaraj, 2013) (India)	PD, CAL	30	30	Mean scoresCAL*- Nontobacco user [4.16 ± 1.13]- Smokeless tobacco user [5.13 ± 0.96]PD*- Nontobacco user [3.49 ± 0.76]- Smokeless tobacco user [4.37 ± 0.77]	
Kathiriya 2016 (Kathiriya et al., 2016) (India)	CPI, CAL	400	400	Unadjusted ORCPI (Score 3-4)* [2.062 (1.55–2.75)]- User: 197/400- Non-user: 128/400CAL [2.234 (1.68-2.98)]	
Kulkarni 2016 (Kulkarni et al., 2016) (India)	PD	121	84	PD > 4mm- SLT: 113/121 - Non-users:38/84	
Mahapatra (Mahapatra et al., 2018) 2018 (India)	PD, LOA	256	256	Unadjusted ORPD* [1.71 (1.19 - 2.48)]- User: 104/256- Non-user: 73/256LOA* [2.39 (1.55 - 3.69)]	
Mittal 2017 (Mittal et al., 2017) (India)	PD, GR	50	50	Mean DifferenceGR*[1.32 ± 0.25]PD [-0.12 ± 0.16]	
Source	Periodontal parameters assesse	Cases	Controls	Summary of main statistical findings	Variables adjusted in statistical analysis
Cross-sectional studies					
Nagarajappa 2010 (Nagarajappa and Prasad, 2010) (India)	CPI, CAL	42	42	CPI > 3 [p > 0.05]- User: 33/42- Non-user: 27/42LOA [p > 0.05]	
Parmar 2008 (Parmar et al., 2008) (India)	PD, GR	168	197	Unadjusted ORPD* > 4mm [1.64 (1.26 - 2.14)] - User: 92/168- Non-user: 61/197GR* [1.72 (1.32 - 2.32)]	
Philip 2013 (Biju Philip et al., 2013) (India)	CPI, LOA	NR	NR	CPI [p = 0.898]LOA [p = 0.631]	
Rajkarnikar 2015 (Rajkarnikar and Acharya, 2014) (Nepal)	PD	25	312	All SLT users (100%) had periodontitis compare to 33.7% in non-users*	
Ravishankar 2017 (Ravishankar PL et al., 2017) (India)	CAL, CPI	100	100	PD (4-5 mm)- User: 46/100- Non-user: 10/100CPI* [p = 0.0001]LOA* [p = 0.0001]	
Shamaz 2013 (Mohamed and Janakiram, 2013) (India)	CAL	641	671	Unadjusted ORPeriodontal disease* [2.18 (1.73 - 2.75)] - User: 316/641- Non-user: 207/671	
Sharma 2018 (Sharma and Oberoi, 2018) (India)	CPITN, LOA	NR	NR	CPI (Score 3,4) [p > 0.05]LOA* (Score 1,2,3) [p < 0.05]	
Singh 2011 (Singh et al., 2011) (India)	CAL, GR, PD, Mob, FI	657	976	Odds RatioCalculus [1.35 (1.126 – 1.616)]GR [3.91 (3.243-4.715)]CAL [3.69 (2.983-4.561)]Mob [3.22 (2.137-4.963)]FI [5.23 (3.013-9.084)]	
Singh 2016 (Singh et al., 2016) (India)	CPI	252	341	CPI* [p < 0.001]PD (> 4mm)- User: 96/252- Non-user: 52/341	
Verma 2019 (Verma et al., 2019) (India)	CAL, GR, Mob and FI	100	100	CAL*: [p = 0.0005]GR*: [p < 0.0001]Mob*: [p = 0.033]FI*: [p < 0.0001]	

Legends, PD, Pocket Depth; CAL, Clinical Attachment Loss; OR, Odds Ratio; GR, Gingival Recession; CPI, Community Periodontal Index; CPITN, Community Periodontal Index and Treatment Needs; LOA, Loss of Attachment; PI, Periodontal Inflammation; Mob, Mobility; MBL, Marginal Bone Loss; FI, Furcation Involvement; * Statistically significant difference between the case and control groups

## Discussion

Effects of SLT on periodontal tissues is the result of deleterious effect of substances or compounds present in tobacco per se (mainly nicotine) and those added before its consumption. Research has shown, nicotine present in SLT causes gingival hyperemia, collagen breakdown and retardation of growth of fibroblasts of gingiva thereby reducing the production of fibronectin and collagen (Mavropoulos et al., 2001; Tipton and Dabbous, 1995). Nicotine is known to affect proper functioning of neutrophils (Pabst et al., 1995). The common substances consumed along with SLT are calcium hydroxide, arecanut and betel quid. The calcium hydroxide or slaked lime increases formation of reactive oxidative species (ROS). The release of these ROS have the potential to damage periodontal tissues (Chapple and Matthews, 2007). Arecanut contains an alkaloid known as “arecoline”, which tends to decrease growth of gingival keratinocytes and fibroblasts causing reduction in collagen synthesis. Arecoline also alter antimicrobial response of neutrophils. Betel quid chewing with or without tobacco is detrimental to periodontal health. Studies had reported increased calculus formation among betelnut chewers, which can lead to destruction of gingival attachment and alveolar bone (Chatrchaiwiwatana, 2006). 

This systematic review was performed to assess the association of SLT consumption with the occurrence of the periodontitis. It is well documented that smoking tobacco use is a major risk factor for periodontitis. Schwendicke F et al. had calculated smoking-attributable burden of periodontal diseases using data from Global Burden of Disease study 2015. It was estimated that the global attributable burden was 251,160 Disability Adjusted Life Years (DALYs) with highest burden in South-East Asia, East Asia and Oceania (83,052 DALYs) regions (Schwendicke et al., 2018). No such data is available for SLT. Assessment of the association of the SLT with periodontitis is important for patient education, to inform policy makers and to strengthen evidence for the clustering of risk factors with Non-Communicable Diseases (NCDs).

The studies included in this systematic review consistently showed the positive association between SLT consumption and periodontitis status. Majority of these studies were cross sectional in nature, hence, even though they had reported a positive association, the temporality could be a major issue. These studies (cross-sectional and case-control studies) are ranked quite low in the evidence-based hierarchy pyramid therefore reporting a conclusive association between SLT and periodontitis based on results of these studies is not appropriate. Previous reviews had also pointed out difficulty in calculating risk of SLT use and its effects on health of human beings (Siddiqi et al., 2015; Critchley and Unal, 2003; Kallischnigg et al., 2008). However, we do feel there is enough merit in testing this hypothesis with methodologically strong studies.

The odds of SLT users having periodontal pockets greater than 4 mm was 3.64 times higher than that of non-users. Similarly, the odds for pocket depth greater than 6 mm was 3.46. Though the odds reported were unadjusted, the statistics provide assumptions worthy regarding the effect of smokeless tobacco on periodontal disease. All included studies had reported a higher PPD among SLT users with reported odds ratio generally ranging from 1.72 to 7.71. Few studies (Baishya et al., 2019; Faiza Amjad et al., 2012; Javed et al., 2013b; Kulkarni et al., 2016; Amjad et al., 2013a and Kulkarni) which reported very high odds ratio were probably due to the very low incidence of outcome among the non-users group and thus may be interpreted with caution. A sensitivity analysis after excluding the above four studies revealed an OR of 2.57 indicating that exclusion of the studies did not cause a significant change in the association.

Gingival recession is another prominent manifestation of periodontal disease. The odds of GR among SLT users were 1.71 times higher. This difference in the strength of association compared to pocket depth could be because pocket formation precedes gingival recession in the progression of periodontal disease. 

Several studies used CPI as an epidemiogical index to measure periodontal disease. Loss of attachment, measured in the CPI index, is a more credible indicator of periodontal disease as an increase in pocket depth could be caused by other factors too (eg: false pocket). The odds of having a LOA of 4-5 mm and 6 mm was 2.83 and 4.05 times higher among SLT users respectively.

Several studies reported periodontal pocket depth, gingival recession and clinical attachment level in terms of mean ± standard deviation. Hence we decided to undertake a quantitative synthesis to compare the difference in means of the parameters. While mean scores were higher in the SLT group, the differences were statistically significant for gingival recession and clinical attachment level. Studies assessing pocket depth reported contrasting results but studies done on larger population (Singh et al., 2011) had significantly higher pocket depth for SLT group. Only data from three studies which reported GR as Mean and SD were included for meta-analysis and all three studies had SLT users with greater recession. The results were similar for CAL which included five studies. 

*Strengths and limitations*

This review is a first attempt to analyze the available evidence on the association between SLT consumption and risk of periodontal disease among the population residing in South East Asian countries. The findings of the current review must be seen in the light of various limitations discussed here. Although we have searched major databases and had a broad search criterion, we cannot confirm that all studies conducted on this topic are included in our review. We might have missed articles published in languages other than English, grey literature, and unpublished reports such as thesis. Second limitation is that except for two (Al-Askar et al., 2017; Al-Tayar et al., 2015), all other included studies in our review were conducted in South Asian countries. However, it is reported that majority of all SLT users in the world are from these countries (Sinha et al., 2012) and SLT used in western world is quite different in composition and mode of consumption from those used in South-East Asian countries, hence their effects on health are not comparable (Critchley and Unal, 2003; Kallischnigg et al., 2008). Another limitation is related to inherent bias present in observational study designs such as selection and recall bias (Khan et al., 2017).

The exposure ascertainment of SLT was also different among the studies. SLT consumption was defined differently in all included the studies. SLT is consumed in various forms which depends on the geographic location and cultural variation of the population. So, the quantity of tobacco present in various forms of SLT may differ for the same frequency and duration. However, considering all forms as SLT products was the rationale behind pooling the results together. The duration of the SLT consumption has been assessed differently ranging from 3 months to 5 years in the included studies. This variation of duration of exposure may reflect the odds ratio estimates mentioned above. The frequency of the SLT consumption also shows wide variation in the included studies. 

Overall, there were high amount of the clinical, methodological and statistical heterogeneity between the studies in assessing the association between the SLT and periodontitis. Hence, the authors recommend to interpret the results of meta-analysis with caution and propose the following recommendations for further research; undertaking studies with a prospective study design with control of confounding factors, inclusion of participants who are consuming SLT for sufficiently long duration, effect of different types of SLT products on periodontitis, analysis on site of placement of SLT in the mouth and using internationally acceptable definition for smokeless tobacco status and periodontitis.

In conclusions, the current systematic review analyzed the quality of evidence probing the association between the SLT consumption and PD with a focus on studies conducted in Asian countries. The strength of association was reported positive by most of the included studies, but the quality of evidence was very low with high methodological heterogeneity. We recommend future studies with standardized methodology in assessing exposure and outcome to obtain precise estimates. 

## Author Contribution Statement

Abhishek Mehta contributed to the design of the review, data acquisition, data interpretation and analysis and drafted the manuscript. Venkitachalam Ramanarayanan and Vineetha Karuveettil contributed to the statistical analysis and drafted the manuscript and Chandrashekar Janakiram contributed to the design of the review, data interpretation and analysis and drafted the manuscript. All authors gave final approval and agree to be accountable to all aspects of the work.
